# Laparoscopic removal of an ingested fish bone that penetrated the stomach and was embedded in the pancreas: a case report

**DOI:** 10.1186/s40792-018-0559-4

**Published:** 2018-12-29

**Authors:** Kosuke Mima, Hidetaka Sugihara, Rikako Kato, Chihiro Matsumoto, Daichi Nomoto, Hironobu Shigaki, Junji Kurashige, Mitsuhiro Inoue, Shiro Iwagami, Takao Mizumoto, Tatsuo Kubota, Nobutomo Miyanari

**Affiliations:** Department of Surgery, National Hospital Organization Kumamoto Medical Center, 1-5 Ninomaru, Chuo-ku, Kumamoto, 860-0008 Japan

**Keywords:** Fish bone, Laparoscopic surgery, Pancreas

## Abstract

**Background:**

The gastrointestinal tract can occasionally be perforated or penetrated by an ingested foreign body, such as a fish bone. However, there are very few reported cases in which an ingested fish bone penetrated the gastrointestinal tract and was embedded in the pancreas.

**Case presentation:**

An 80-year-old male presented with epigastric pain. Computed tomography of the abdomen showed a linear, hyperdense, foreign body that penetrated through the posterior wall of the gastric antrum. There was no evidence of free air, abscess formation, migration of the foreign body into the pancreas, or pancreatitis. As the patient had a history of fish bone ingestion, we made a diagnosis of localized peritonitis caused by fish bone penetration of the posterior wall of the gastric antrum. We first attempted to remove the foreign body endoscopically, but failed because it was not detected. Hence, an emergency laparoscopic surgery was performed. A linear, hard, foreign body penetrated through the posterior wall of the gastric antrum and was embedded in the pancreas. The foreign body was safely removed laparoscopically and was identified as a 2.5-cm-long fish bone. Intraperitoneal lavage was performed, and a drain was placed in the lesser sac. The patient recovered without complications and was discharged on the 7th postoperative day.

**Conclusion:**

Laparoscopic surgery could be performed safely for the removal of an ingested fish bone embedded in the pancreas.

## Introduction

The gastrointestinal tract can occasionally be perforated or penetrated by ingested foreign bodies, such as fish bones, press-through packages, dental plates, and needles [1, 2]. Although most ingested foreign bodies pass through the gastrointestinal tract uneventfully, approximately 10 to 20% require endoscopic removal and approximately 1% require surgery [3]. There are very few reported cases in which an ingested foreign body penetrated the gastrointestinal tract and was embedded in the pancreas. We herein report a case of laparoscopic removal of an ingested fish bone that was embedded into the pancreas.

## Case presentation

An 80-year-old male with hypertension and chronic kidney disease was admitted to our hospital because of epigastric pain that had begun after dinner 1 day before admission. The patient reported no use of nonsteroidal anti-inflammatory drugs.

**Fig. 1:**
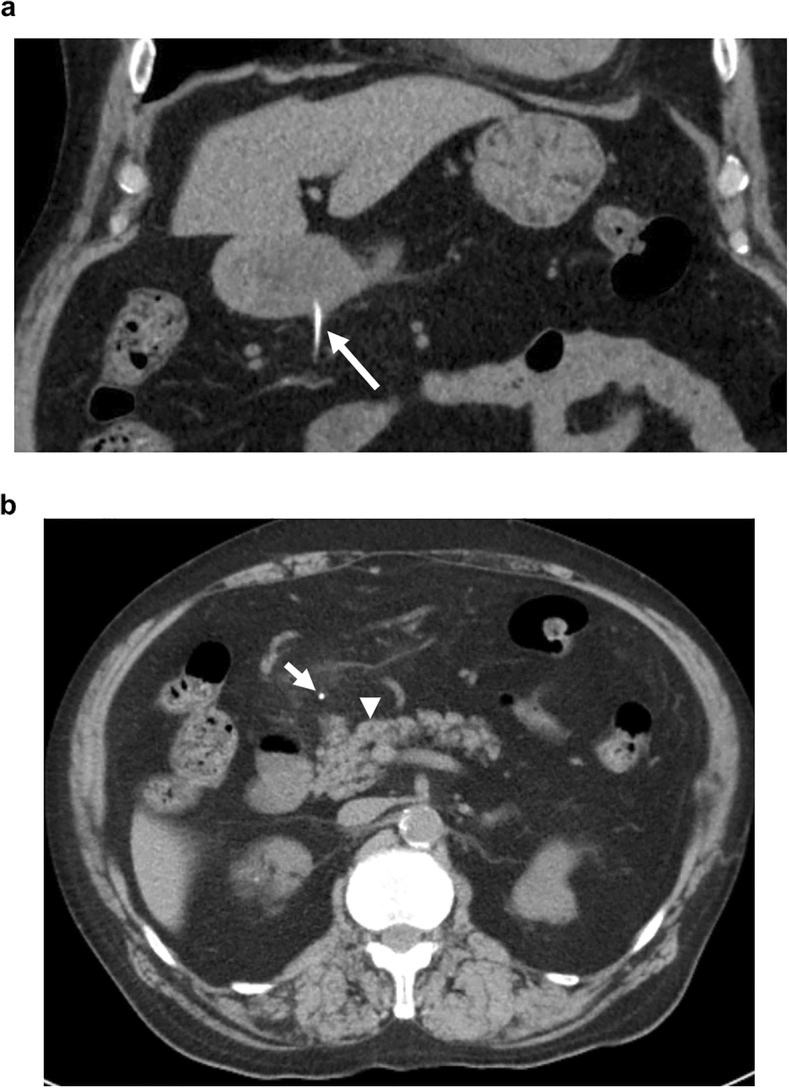
Coronal view of a computed tomography (CT) image showing a linear, hyperdense, foreign body (arrow), which appeared to penetrate through the posterior wall of the gastric antrum (**a**). CT image showing the foreign body (arrow) and the pancreas (arrowhead) (**b**)

On examination, the patient’s temperature was 37.0 °C, heart rate was 101 beats per minute, blood pressure was 185/93 mmHg, respiratory rate was 18 breaths per minute, and oxygen saturation was 96% while the patient was breathing ambient air. The patient’s body mass index was 26.3 kg/m^2^. Mild epigastric tenderness was present. The remainder of the physical examination was normal. The laboratory data on admission were as follows: white blood count 9400/mm^3^, the C-reactive protein level 7.53 mg/dl, red blood count 419 × 10^4^/mm^3^, hemoglobin 13.4 g/dl, platelets 16.8 × 10^4^/mm^3^, total bilirubin 0.8 mg/dl, aspirate aminotransferase 21 IU/l, alanine aminotransferase 16 IU/l, alkaline phosphatase 232 IU/l, γ-glutamyl transferase 15 IU/l, amylase 86 IU/l, blood urea nitrogen 32 mg/dl, and creatinine 1.99 mg/dl, and estimated glomerular filtration rate is 26 ml per minute per 1.73 m^2^ of body surface area. Chest and abdominal radiography showed no abnormalities. Computed tomography (CT) of the abdomen showed a linear, hyperdense, foreign body that appeared to penetrate through the posterior wall of the gastric antrum (Fig. 1a). There was no evidence of free air, abscess formation, migration of the foreign body into the pancreas, or pancreatitis (Fig. 1b).

**Fig. 2:**
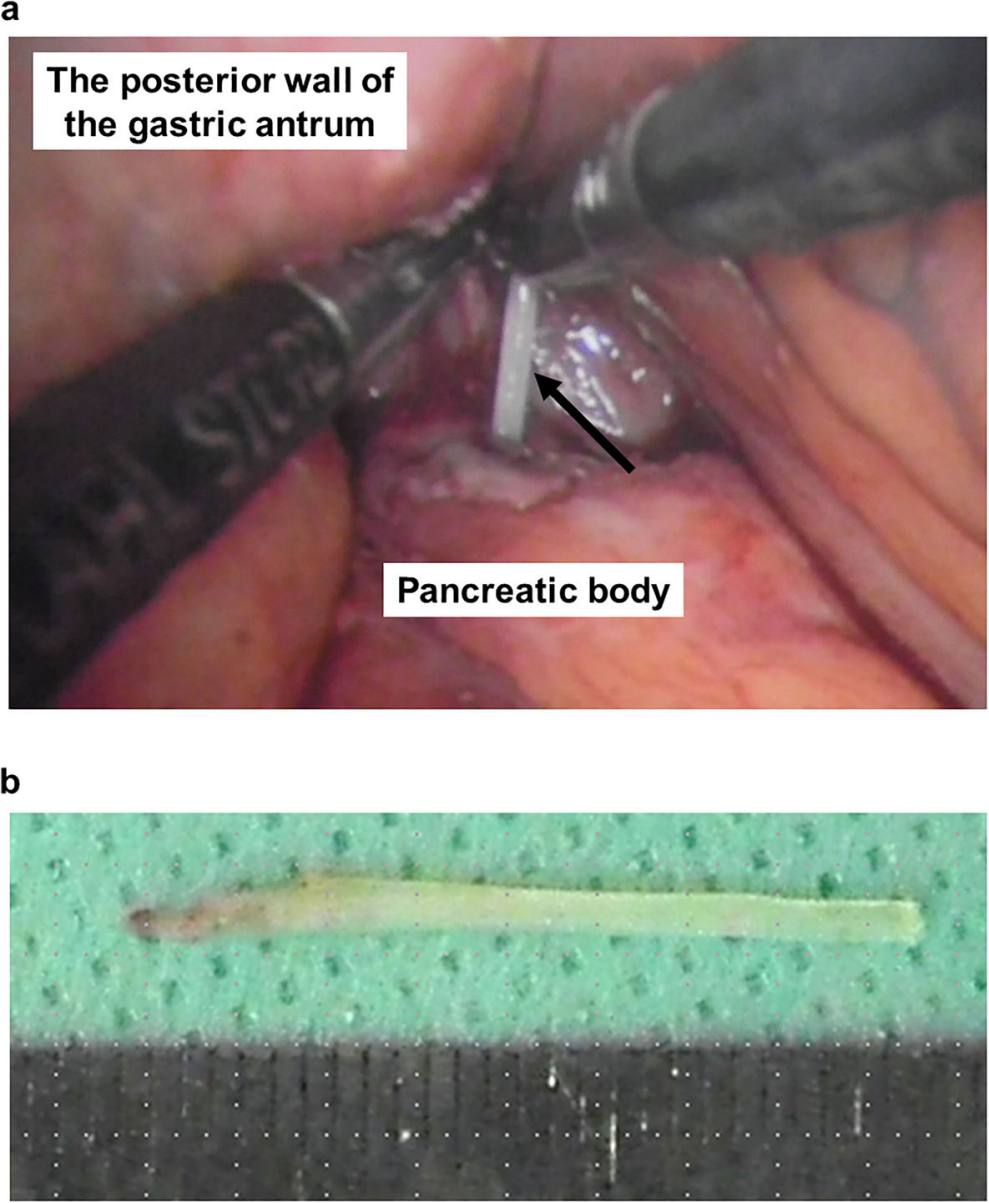
A linear, hard, foreign body (arrow) was found in the adhesive tissue between the gastric antrum and the pancreatic body (**a**). A photograph taken immediately after removal of the specimen showing that it was a 2.5-cm-long fish bone (**b**)

As the patient had a history of fish bone ingestion, we made a diagnosis of localized peritonitis caused by fish bone penetration of the posterior wall of the gastric antrum. We first attempted to remove the foreign body endoscopically, but failed because it was not detected. Hence, an emergency laparoscopic surgery was performed. The patient was placed in a supine position. The operator stood on the left side of the patient, the assistant on the right side, and the scopist between the patient’s legs. Four trocars were placed: one above the navel for the laparoscopy (12 mm), two in the upper left abdominal quadrant (5 mm), and one in the upper right abdominal quadrant (5 mm). Laparoscopic gastrectomy techniques were used to separate the greater omentum from the transverse colon and open the lesser sac. A linear, hard, foreign body was found in the adhesive tissue between the gastric antrum and the pancreatic body (Fig. 2a). The foreign body was safely removed from both the pancreas and stomach laparoscopically. The foreign body was identified as a 2.5-cm-long fish bone, (Fig. 2b). The length of the fish bone embedded in the pancreas was about 2 cm. There was a minor laceration at the site of the pancreatic injury. Neither fat saponification nor hematoma around the pancreas was identified. We did not perform suture repair or local debridement for the site of the pancreatic injury. Because the site of the penetrated gastric wall was small and a leak was not observed, we did not perform suture repair or cover the site of the penetrated gastric wall with the omentum. Intraperitoneal lavage was performed, and a drain was placed in the lesser sac.

Clear fluid was drained, and the postoperative serum amylase levels were normal. The patient recovered without complications and was discharged on the seventh postoperative day.

## Discussion

Fish bones are one of the most common ingested foreign bodies [4]. The most common sites of perforation are the terminal ileum, sigmoid colon, and rectum [5]. We searched PubMed for English literature reporting cases of an ingested fish bone that was embedded in the pancreas, using the terms “fish bone” and “pancreas.” The review of the English literature revealed only six cases of an ingested fish bone that penetrated through the gastrointestinal tract and migrated into the pancreas [6–11]. In these cases, a fish bone penetrated the stomach [6, 7, 10] or the duodenum [8, 9, 11].

Preoperative diagnosis of perforation of the gastrointestinal tract by an ingested foreign body is difficult, as patients usually cannot recall any recent history of foreign body ingestion. CT is useful for detecting an ingested fish bone and its associated complications [1]. CT often reveals a linear, hyperdense, foreign body corresponding to a bone. We were able to make an accurate preoperative diagnosis of fish bone penetration of the stomach, based on the history of ingestion of a fish bone and the CT findings. However, unlike in previously reported cases, CT in the present case showed no evidence that the foreign body was embedded in the pancreas. Hence, it is necessary to evaluate for pancreatic injury when a fish bone penetrates the stomach or duodenum, even if there are no CT findings of migration of the foreign body into the pancreas.

The treatment for penetration of the gastrointestinal tract by an ingested fish bone consists of endoscopic removal, surgical removal, abscess drainage if necessary, and administration of appropriate antibiotics. If pancreatic injuries are suspected, surgical removal of an ingested fish bone may be required to evaluate the pancreas and manage pancreatic injuries [12]. In all reported cases, the fish bone embedded in the pancreas was removed by laparotomy. To the best of our knowledge, there are very few reported cases of laparoscopic removal of a fish bone embedded in the pancreas. We could safely remove the fish bone and perform intraperitoneal lavage and drainage laparoscopically.

An endoscopic removal has also been shown to be effective in the management of an ingested foreign body [13]. If there was no evidence of free air, abscess formation, migration of the foreign body into the pancreas, or pancreatitis, an endoscopic examination would be attempted first not only for the diagnosis but also for the removal of the detected foreign body. In the present case, we first attempted to remove the foreign body endoscopically, but failed because it was not detected.

In summary, the present case demonstrates an unusual presentation of an ingested fish bone that penetrated the gastric antrum and migrated to the pancreas; this fish bone was successfully removed laparoscopically. Laparoscopic surgery could be performed safely for the removal of an ingested fish bone embedded in the pancreas.

## Abbreviation

CT: Computed tomography

## References

[1] Guelfguat M, Kaplinskiy V, Reddy SH, DiPoce J (2014). Clinical guidelines for imaging and reporting ingested foreign bodies. AJR Am J Roentgenol.

[2] Jain A, Nag HH, Goel N, Gupta N, Agarwal AK (2013). Laparoscopic removal of a needle from the pancreas. J Minim Access Surg.

[3] Birk M, Bauerfeind P, Deprez PH, Hafner M, Hartmann D, Hassan C (2016). Removal of foreign bodies in the upper gastrointestinal tract in adults: European Society of Gastrointestinal Endoscopy (ESGE) Clinical Guideline. Endoscopy.

[4] Kim HU (2016). Oroesophageal fish bone foreign body. Clin Endosc.

[5] McCanse DE, Kurchin A, Hinshaw JR (1981). Gastrointestinal foreign bodies. Am J Surg.

[6] Goh BK, Jeyaraj PR, Chan HS, Ong HS, Agasthian T, Chang KT (2004). A case of fish bone perforation of the stomach mimicking a locally advanced pancreatic carcinoma. Dig Dis Sci.

[7] Wang WL, Liu KL, Wang HP (2008). Clinical challenges and images in GI. Pancreatic abscess resulting from a fish bone penetration of the stomach. Gastroenterology.

[8] Yasuda T, Kawamura S, Shimada E, Okumura S (2010). Fish bone penetration of the duodenum extending into the pancreas: report of a case. Surg Today.

[9] Symeonidis D, Koukoulis G, Baloyiannis I, Rizos A, Mamaloudis I, Tepetes K (2012). Ingested fish bone: an unusual mechanism of duodenal perforation and pancreatic trauma. Case Rep Gastrointest Med.

[10] Huang YH, Siao FY, Yen HH (2013). Pre-operative diagnosis of pancreatic abscess from a penetrating fish bone. QJM.

[11] Gharib SD, Berger DL, Choy G, Huck AE. CASE RECORDS of the MASSACHUSETTS GENERAL HOSPITAL. Case 21-2015. A 37-year-old American man living in Vietnam, with fever and bacteremia. N Engl J Med 2015;373:174–183. 10.1056/NEJMcpc1411439 26154791

[12] Debi U, Kaur R, Prasad KK, Sinha SK, Sinha A, Singh K (2013). Pancreatic trauma: a concise review. World J Gastroenterol.

[13] Sugawa C, Ono H, Taleb M, Lucas CE (2014). Endoscopic management of foreign bodies in the upper gastrointestinal tract: a review. World J Gastrointest Endosc.

